# Porcine genetic markers of immunocompetence shape the immune response to a classical swine fever virus subunit vaccine

**DOI:** 10.3389/fcimb.2026.1803004

**Published:** 2026-08-11

**Authors:** Joaquim Tarrés, Carles Hernández-Banqué, Llilianne Ganges, Teodor Jové-Juncà, Olga González-Rodríguez, Liani Coronado, Yumel Sordo-Puga, Sofia Gol, Josep Reixach, Raquel Quintanilla, Maria Ballester

**Affiliations:** 1 Institute of Agrifood Research and Technology (IRTA), Animal Breeding and Genetics, Barcelona, Spain; 2 Institute of Agrifood Research and Technology (IRTA), Animal Health, CReSA, Universitat Autònoma de Barcelona (UAB), Barcelona, Spain; 3 WOAH Reference Laboratory for Classical Swine Fever, Institute of Agrifood Research and Technology (IRTA), CReSA, Barcelona, Spain; 4 Departamento de Salud Animal, Centro de Ingeniería Genética y Biotecnología, Havana, Cuba; 5 Seleccion Batalle SA, Girona, Spain

**Keywords:** CRP, genotyping, immunogenetics, neutralizing antibodies, pig, SNP, γδ T cells

## Abstract

Breeding programs aimed at enhancing robustness and disease resistance in pigs represent a complementary strategy to biosecurity and vaccination measures. The objective of the present study was to validate a panel of genetic biomarkers and immunity phenotypes as indicators of the immune response to the E2-CD154 subunit vaccine against classical swine fever virus (CSFV). A total of 120 eight-week-old Duroc pigs were genotyped using a panel of six genetic markers, previously described as associated with innate and adaptive immunity traits. Additionally, total plasmatic IgG concentrations and *SOX13* gene expression levels in blood were quantified. Based on these parameters, two genetically divergent groups (FAV and UNFAV) comprising 13 pigs each were selected and vaccinated twice on days 0 and 21 with the commercial vaccine. Serum samples were collected prior to vaccination and on days 14, 21, 28 and 35 post-vaccination. CSFV-specific antibody measures were assessed by ELISA targeting the E2 glycoprotein and by virus seroneutralization tests against three different CSFV strains. Vaccination with the E2-CD154 subunit vaccine induced strong humoral responses, with all animals developing high CSFV-E2–specific antibody levels by 14 days post-vaccination. The FAV group showed significantly higher ELISA and neutralizing antibody (NAb) titers than the UNFAV group, particularly at 21 days. After the second vaccine dose, all pigs reached high NAb titers, although group differences persisted for some CSFV strains. Animals in the FAV group exhibited higher frequencies of the A allele of SNP rs342772739 (associated with elevated γδ T cell counts and increased *SOX13* expression), which, together with the G allele of SNP rs713631040 (associated with reduced serum C-reactive protein levels), showed the strongest association with NAb responses. Additional SNPs, rs80803525, rs319560097, rs338661853 and rs1113630513, also showed significant or suggestive associations with strain- and time-dependent variation in NAb responses. Our findings demonstrate the existence of genetic variability influencing the immune response to CSFV vaccination and identify a set of candidate genetic biomarkers associated with vaccine-induced humoral immunity in pigs.

## Graphical Abstract

**Figure:**
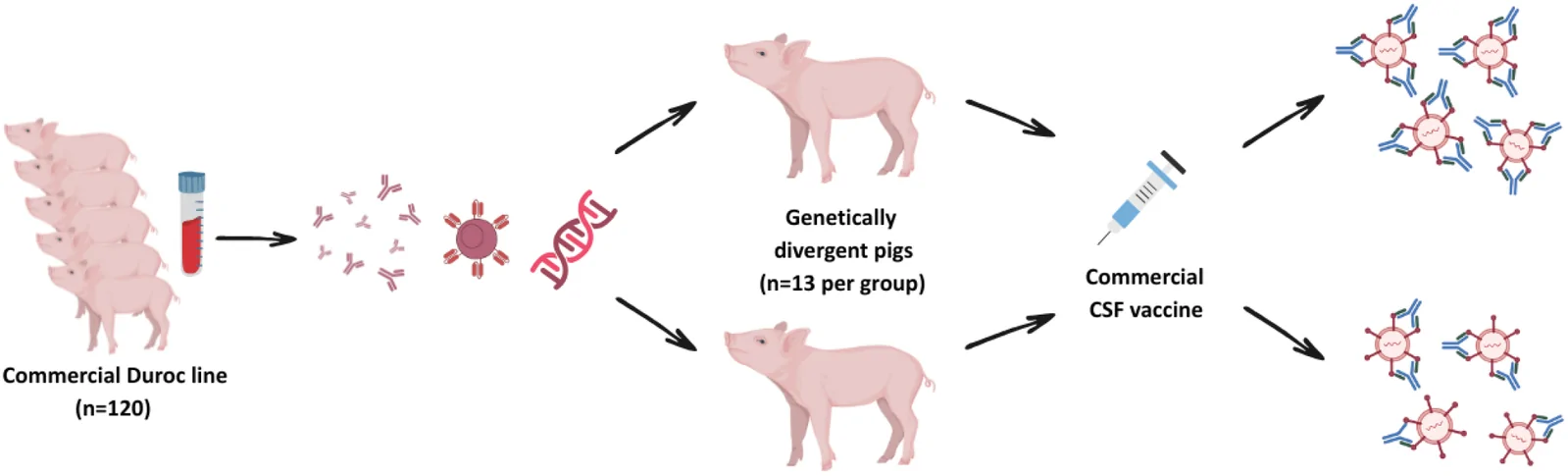

## Introduction

The intensification of pig production systems, combined with the global trade of pigs and pork products and the environmental changes driven by climate change, increases the likelihood of pathogen emergence, re-emergence, and spread. Under these circumstances, the genetic improvement of animal populations to enhance their robustness and disease resistance represents a valuable complement to traditional control measures such as biosecurity, vaccination, and therapeutic interventions (Bai and Plastow, 2022). Previous studies have established the genetic basis of health-related traits (Edfors-Lilja et al., 1994; Henryon et al., 2006; Clapperton et al., 2008, 2009; Flori et al., 2011; Ballester et al., 2020, 2023), supporting the inclusion of these traits or their associated genetic markers in pig breeding programs to enhance the overall immunocompetence of animals and facilitate the selection of animals for disease resilience against a wide variety of pathogens. Within this framework, a previous study allowed us identifying a panel of genetic markers associated with innate and adaptive immunity traits (Ballester et al., 2020, 2023; Hernández-Banqué et al., 2023), which were linked to pig survival during a PRRSV outbreak (Tarrés et al., 2024). Blood γδ T cells and IgG levels were selected as relevant phenotypes since they represent key and complementary components of the immune system. γδ T cells constitute a prominent lymphocyte population in porcine peripheral blood and possess the potential to combine both conventional adaptive and innate-like responses (Takamatsu et al., 2006; Vantourout and Hayday, 2013). In contrast, IgG levels reflect humoral adaptive immunity and are commonly used as indicators of immunological memory and long-term humoral immunity (Manz et al., 2005). Genetic markers associated with other humoral-related traits, including the phagocytic capacity of lymphocytes and memory T-helper cells, were also identified (Ballester et al., 2020, 2023). B cells have been shown to efficiently phagocytose particulate antigens and present them to CD4^+^ T cells more effectively than soluble antigens, thereby enhancing humoral immune responses (Martínez‐Riaño et al., 2018). Likewise, the induction of memory T cells is considered a major determinant of long-term vaccine efficacy, as these cells mediate durable cellular immunity and contribute to rapid recall responses following antigen re-exposure (Ura et al., 2022). Finally, a functionally validated marker associated with blood C-reactive protein (CRP) levels was also identified (Hernández-Banqué et al., 2023). C-reactive protein (CRP) is a marker of systemic inflammation and a regulator of innate immune activation and inflammatory progression (Yao et al., 2019). Additionally, CRP binds to Fcγ receptors on leukocytes, increasing IgG production and secondarily influencing the adaptive immune response (Yao et al., 2019; Khalil and Al-Humadi, 2020).

Classical swine fever (CSF) is a highly contagious viral disease that poses a major threat to the global swine industry, with serious economic and sanitary consequences, and is therefore notifiable to the World Organisation for Animal Health (WOAH) (Ganges et al., 2020). Classical swine fever virus (CSFV), the etiological agent of the disease, belongs to the genus Pestivirus within the family Flaviviridae. CSFV is an enveloped virus containing a positive-sense, single-stranded RNA genome (Postel et al., 2021).

The WOAH recommends the use of marker vaccines capable of differentiating infected from vaccinated animals (DIVA concept), an approach that has the potential to mitigate the negative impact of CSF on international trade and animal welfare. The E2-CD154 chimeric recombinant protein, consisting of the CSFV E2 glycoprotein fused to the porcine CD154 molecule, constitutes the active component of the Porvac^®^ subunit vaccine (Sordo-Puga et al., 2021). When administered parenterally, Porvac^®^ confers solid protection against CSFV and has proven effective in controlling field outbreaks (Sordo-Puga et al., 2021, 2025; Suárez-Pedroso et al., 2021). The E2 glycoprotein is the most immunogenic CSFV protein (Ganges et al., 2020). Neutralizing antibodies directed against the E2 are produced between 10 and 20 days after natural infection or vaccination with E2-based vaccines (van Rijn et al., 1994) and represent the only antibodies capable of conferring clinical protection against CSFV challenge (van Rijn et al., 1996). Furthermore, the presence of IFN-γ–secreting cells, including T cells and natural killer (NK) cells, has been detected and associated with the immune mechanisms mediating vaccine-induced protection (Tarradas et al., 2010; Auray et al., 2020; Ganges et al., 2020; Chen et al., 2024; Coronado et al., 2025). However, some innate and early adaptive immune mechanisms involved in protection against CSFV remain poorly understood.

The aim of this study was to validate a set of genetic biomarkers and immune phenotypes associated with both innate and adaptive immune mechanisms as indicators of the immune response in pigs, using vaccination against CSFV as an experimental model. To achieve this goal, we compared the quantitative and qualitative CSFV-specific antibody responses, including neutralizing activity, between two groups of pigs genetically divergent for selected immunity-related traits and genetic markers.

## Materials and methods

### Animal material

A total of 120 female pigs from a commercial Duroc pig line were used for this study. The animals originated from 74 litters, produced by 74 sows and 19 boars, and were organized in 6 pens with 20 animals per pen. All pigs were raised on the same farm under uniform management conditions and fed *ad libitum* with a commercial cereal-based diet. At the time of sampling, all animals appeared healthy and showed no clinical signs of infection.

Blood samples were collected for all pigs at eight weeks of age via the external jugular vein using vacutainer tubes containing heparin or EDTA (Sangüesa S.A., Spain) and Tempus™ Blood RNA tubes (Thermo Fisher Scientific, Spain) to stabilize the RNA. All samples were transported to the laboratory with ice blocks and stored at -20°C or -80°C until further processing.

### Phenotypic analysis

The 120 female pigs were screened for immunity parameters and biomarkers as previously detailed in Tarrés et al. (2024). Total plasma IgG concentration was determined using a commercial ELISA kit (Bethyl laboratories Inc., Bionova, Spain), following the manufacturer’s protocol. Plasma was collected from blood sampled in 6 ml heparinised tubes and diluted 1:50,000. Each sample was analyzed in duplicate, and IgG concentrations were calculated by interpolating absorbance values against standard curves generated with known concentrations of porcine IgG, accounting for the applied dilution factor. Absorbance was read at 450 nm using a microplate reader (LUMistar Omega, BMG Labtech, Germany) and processed with Omega MARS software (BMG Labtech, Germany).

The expression of *SOX13* gene, a key transcription factor in γδ T-cell development, was quantified by qPCR. Total RNA was extracted from whole blood collected in Tempus™ tubes using the Tempus™ Spin RNA Isolation Reagent Kit (Thermo Fisher Scientific, Spain). RNA quantity and purity were evaluated with a Nanodrop ND-1000 spectrophotometer. One μg of RNA was reverse transcribed into cDNA using the PrimeScript RT Reagent Kit (Takara, Condalab, Spain) in a 20 μl reaction volume, according to the manufacturer’s instructions.

Primers for *SOX13* (F-5’-AAGCCAAAGACGTCAAAGGGA-3’ and R-5’-TCCCGAAGGGTGGACAGTT-3’) were designed using PrimerExpress 2.0 software (Applied Biosystems). The reference genes *β2M* and *HPRT1* were used for normalization (Ballester et al., 2017). Quantitative PCR was performed on a QuantStudio™ 12 K Flex Real-Time PCR System (Thermo Fisher Scientific) using SYBR Green chemistry (SYBR™ Select Master Mix, Thermo Fisher Scientific, Spain). Each reaction contained 15 μl total volume, including 3.75 μl of 1:20 diluted cDNA and 300nM of each primer. The thermal cycle was: 10 min at 95 °C, 40 cycles of 15 sec at 95 °C and 1 min at 60 °C. All samples were analysed in duplicate. Data was analysed using the Thermo Fisher Cloud software (Applied Biosystems) and the comparative Ct method (Schmittgen and Livak, 2008), using the sample with the lowest *SOX13* expression as the calibrator.

### Genetic analysis

A panel of six genetic markers (**Table 1**) associated with plasma IgG levels (rs319560097), serum CRP levels (rs81233340), the percentages of γδ T cells (rs342772739) and memory T-helper cells (rs1113630513), lymphocyte counts in blood (rs80803525) and the phagocytic capacity of lymphocytes (rs338661853) (Ballester et al., 2020, 2023) was genotyped in all 120 pigs. Genomic DNA was extracted from EDTA-collected blood samples using a chemagic 360 instrument with the DNA blood 250 Kit H96 (PerkinElmer, Baesweiler, Germany). DNA concentration and purity were measured with a Nanodrop ND-1000 spectrophotometer. Genotyping was performed using KASP methodology on a QuantStudio™ 12 K Flex Real-Time PCR System (Thermo Fisher Scientific).

**Table 1:** List of genetic markers genotyped in the pig population.

Ensembl ID	Alleles	Associated trait
rs338661853	G/A	Phagocytic capacity of lymphocytes
rs319560097	C/T	IgG concentration in plasma
rs80803525	T/C	Lymphocytes count
rs342772739	G/A	Abundance of γδ T cells
rs713631040	A/G	C-reactive protein concentration in serum
rs1113630513	C/T	Relative abundance of T memory cells

Association tests between the immunity phenotypes (IgG levels and *SOX13* expression) and genotypes for the genetic markers were performed using Student’s t-test for each genetic marker genotype. All statistical analyses were performed using version 4.2.2 of the R software (R Core Team, 2020), and statistical significance was defined as *P* < 0.05.

### Genetic index for immune response

A vaccine immune response index value was defined and calculated for each pig *i* by summing the alleles of each genetic marker as follows:


indexi=∑k sik

where s_ik_ is the animal genotype (coded as 0,1,2) for the k^th^ SNP. The six genotyped markers (**Table 1**) were considered to generate the index. A value of 1 was assigned to the T allele of SNP rs319560097 (increased plasma IgG), the A allele of SNP rs342772739 (elevated γδ T cell counts and *SOX13* expression), the A allele of SNP rs338661853 (elevated phagocytic capacity of lymphocytes), the G allele of SNP rs713631040 (reduced serum C-reactive protein levels), the C allele of SNP rs80803525 (lower lymphocyte counts) and the C allele of SNP rs1113630513 (higher T memory cells) (Ballester et al., 2020, 2023). A value of 0 was assigned for the other alleles. Considering all this information, two groups of 13 pigs were selected based on the vaccine immune response index. Animals were classified as favourable (FAV) when their index values ranged from 4 to 9, and as unfavourable (UNFAV) when their values ranged from 0 to 3.

### Experimental design and vaccination schedule

**Figure 1:**
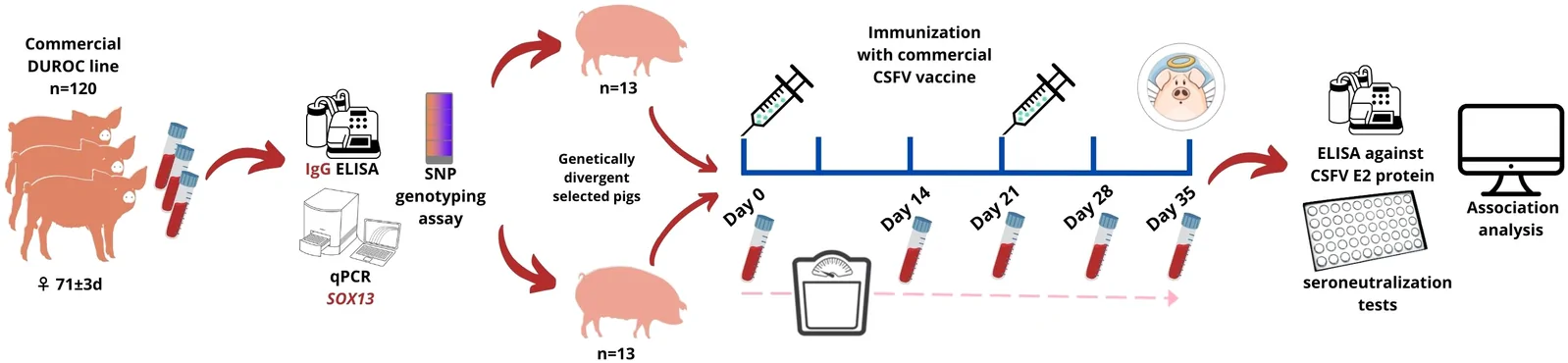
Graphical abstract of the materials and methods used in the CSFV vaccination experiment.

Two groups of 13 ten-week-old pigs, genetically divergent based on the vaccine immune response index value, were housed under biosafety level 2 conditions at the IRTA Pig Experimental Farm (Monells, Girona). The pigs stayed in 4 pens (six animals per pen) and fed *ad libitum* with a commercial cereal-based diet (code D392T172; La Gironina SCOOP, Spain). The ingredient and nutrient composition of diets are shown in **Supplementary Table 1**. Following a seven-day acclimation period, all animals were vaccinated twice, on days 0 and 21, by intramuscular injection in the neck with the standard 2 mL dose of the commercial Porvac^®^ vaccine, containing 50 µg of E2-CD154 antigen (25 µg/mL) formulated in the oily adjuvant Montanide ISA 50 V2 (SEPPIC, France). Blood samples were collected from the external jugular vein using vacutainer tubes without anticoagulants prior to vaccination and on days 14, 21 (before booster vaccination), 28 and 35 post-vaccination. The blood samples were kept at room temperature for 2 h and then stored overnight at 2-8 °C to allow serum extraction. Animals were weighed weekly from the beginning until the end of the experiment. **Figure 1** shows the timeline of the study, including the number of animals used and samples collected.

### CSFV-specific and neutralizing antibodies detection

CSFV-specific antibodies were measured by ELISA against the CSFV E2 glycoprotein using a commercial kit (CSFV Ab test, IDEXX Laboratories, Liebefeld, Switzerland). Results were expressed as blocking percentage values, and serum samples with values ≥40% were considered positive for CSFV E2-specific antibodies. Neutralizing antibodies were quantified using the neutralization peroxidase-linked assay (NPLA) (Terpstra et al., 1984). Serum samples were evaluated for their ability to neutralize cell culture-adapted CSFV reference strains, including Alfort/187, Margarita, and Diepholz. Neutralizing antibody titers (NAb) were defined as the reciprocal of the highest serum dilution capable of neutralizing 100 TCID_50_ of each CSFV strain in 50% of the replicate cell cultures. Animals were considered positive for neutralizing activity when titers were ≥1:5 (Terpstra et al., 1984; Wang et al., 2022). Neutralizing responses were summarized as geometric mean titers (GMT) for each CSFV strain, together with their corresponding confidence intervals.

The normality of the ELISA and neutralizing antibody data was assessed using the Shapiro–Wilk test. Differences in CSFV-specific antibody titers between the two experimental groups were evaluated using Welch’s t-test. All statistical analyses were performed using version 4.2.2 of the R software (R Core Team, 2020), and statistical significance was defined as *P* < 0.05.

### Combined neutralizing antibody response

To integrate neutralizing antibody (NAb) responses against the three CSFV strains, seroneutralization data collected at days 14, 21, 28, and 35 post-vaccination were treated as discrete variables and classified into six categories according to the highest NAb titer observed among the CSFV strains: negative (0), very low response (<1:20), low to moderate response (1:20 to <1:40), moderate response (1:40 to <1:80), high positive response (1:80 to <1:160), and strong positive response (≥1:160). Association tests between NAb responses and the experimental genetic groups were performed using a grouped data model (Prentice and Gloeckler, 1978). In this model, it is assumed that the underlying continuous NAb response is only observed in disjoint discrete intervals [1=a1,a2), 2=[a2,a3),…,k=[ak,ak+1=∞). The probability of neutralizing in the j-th interval for animal i is:

$prob\left\{Y\in \left[u_{j},u_{j+1}\right)\right\}=prob\left\{Y\geq u_{j}\right\}-prob\left\{Y\geq u_{j+1}\right\}=S{\left(u_{j}\right)}^{exp\left(a_{k}\right)}-S{\left(u_{j+1}\right)}^{exp\left(a_{k}\right)}$ where the survivor function at the start of the jth interval $S\left(u_{j}\right)$ is elevated to the exponential of the allele substitution effect a_k_ of the k^th^ genetic group s_ik_ (coded as 0,1). All statistical analyses were performed using version 4.2.2 of the R software (R Core Team, 2020), and statistical significance was defined as *P* < 0.05.

## Results

### Immune-related phenotypes and genetic marker distribution

Mean total plasma IgG concentration and *SOX13* mRNA expression levels in blood of 120 female pigs from a commercial Duroc line, were 4.91 ± 1.14 mg/ml for IgG and 3.58 ± 1.82 for *SOX13* expression. **Table 2** shows the allelic frequencies of the six immunity-associated markers, as well as their associations with IgG levels and *SOX13* gene expression in these animals. The minor allele A for SNP rs342772739 was associated with higher *SOX13* gene expression levels. The minor allele T for SNP rs319560097 was also associated with higher IgG plasma levels and higher *SOX13* gene expression levels. The other SNPs were not associated neither with IgG plasma levels nor *SOX13* gene expression levels.

**Table 2:** Allele frequencies for the SNPs genotyped in the 120 animals and substitution effect of the minor allele on plasma IgG and *SOX13* gene expression levels in blood.

SNP Ensembl ID	Allele (frequency)		Substitution effect	
	Major	Minor	IgG	SOX13
rs338661853	G (0.82)	A (0.18)	-0.07	0.07
rs319560097	C (0.61)	T (0.39)	0.25 *	0.49 *
rs80803525	T (0.58)	C (0.42)	0.07	0.13
rs342772739	G (0.84)	A (0.16)	0.08	0.52 *
rs713631040	A (0.91)	G (0.09)	-0.13	-0.37
rs1113630513	C (0.63)	T (0.37)	-0.03	0.23

^*^Estimates within a SNP row were significantly different at a nominal P<0.05.

Animals from both FAV and UNFAV groups showed similar plasma IgG concentration (4.57 mg/ml for FAV group and 4.55 mg/ml for UNFAV group); whereas the *SOX13* mRNA expression level in blood was significantly higher in the FAV group compared with the UNFAV group, with mean values of 4.81 for FAV group and 3.24 for UNFAV group (*P* < 0.05).

Animals classified within the FAV group exhibited a higher frequency of specific alleles previously associated with immune-related traits. These included the A allele of rs342772739, linked to increased γδ T cell abundance and elevated *SOX13* gene expression; the G allele of rs713631040, associated with reduced serum CRP levels; the T allele of rs319560097, correlated with higher plasma IgG concentrations; the C allele of rs80803525, related to lower blood lymphocyte counts; the C allele of rs1113630513, associated with an increased proportion of memory T cells and the A allele of rs338661853, associated with increased phagocytic capacity of lymphocytes.

### Serum antibody response against CSFV E2 protein

**Figure 2:**
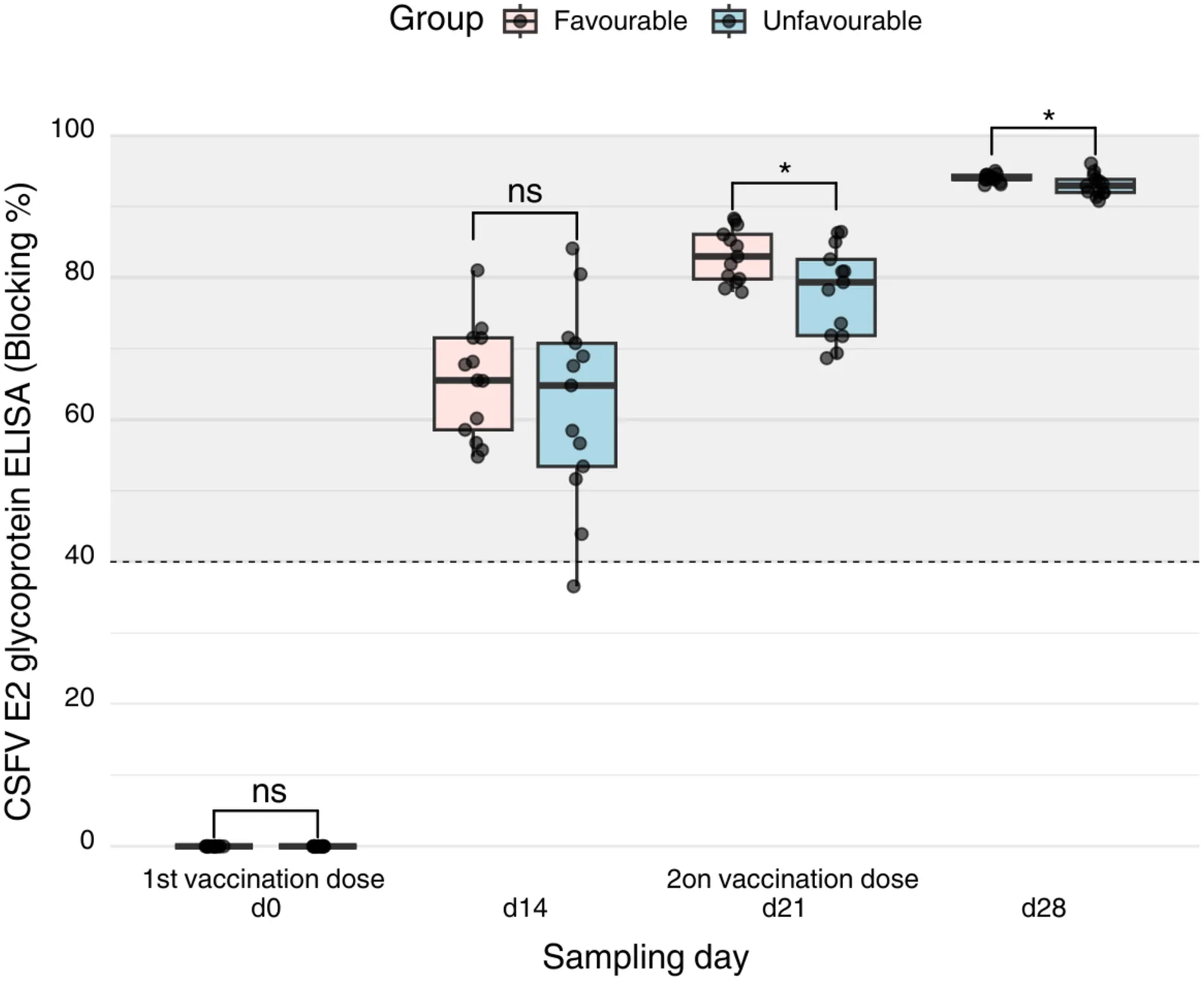
Antibody responses generated in both animal groups (FAV and UNFAV) after E2-CD154 vaccination. Serum samples from FAV pigs (dots in the pink boxplot) and UNFAV pigs (dots in the blue boxplot) were evaluated for the presence of antibodies against E2 glycoprotein using a commercial ELISA. Results are expressed as % blocking, with values above 40% (grey-shaded area) considered positive. *P < 0.05.

All animals vaccinated with the Porvac^®^ E2-CD154 subunit vaccine developed high levels of CSFV-E2-specific antibodies by day 14 post-vaccination following the first vaccine dose (**Figure 2**). Significantly higher CSFV E2-specific antibody levels were observed in the FAV group compared with the UNFAV group at 21-days post-vaccination. After administration of the second vaccine dose, ELISA antibody levels continued to increase in both groups at 28 days post-vaccination, with the FAV group exhibiting significantly higher titers than the UNFAV group.

### Neutralizing antibody response

The NAb response was evaluated in all animals against three different CSFV strains, revealing substantial strain-dependent variability in response magnitude (**Table 3**).

**Table 3:** Neutralizing antibody response against the three different CSFV strains (Alfort/187, Margarita, and Diepholz) evaluated in the 13 animals of both the favourable and unfavourable genetic groups.

Genetic group		CSFV Alfort strain				CSFV Diepholz strain				CSFV Margarita strain			
	Pigs ID	14dpv	21dpv	28dpv	35dpv	14dpv	21dpv	28dpv	35dpv	14dpv	21dpv	28dpv	35dpv
UNFAVOURABLE	47	-	1/10	1/160	1/160	-	1/20	1/1280	1/640	-	-	1/2560	1/2560
	73	1/10	1/20	1/160	1/320	-	1/10	1/2560	1/1280	1/10	-	1/2560	1/1280
	109	-	-	1/80	1/320	-	1/40	1/1280	1/1280	-	1/10	1/5120	1/10240
	112	1/10	-	1/320	1/640	-	1/10	1/1280	1/5120	1/10	1/20	1/2560	1/10240
	4	-	-	1/80	1/80	1/10	1/40	1/1280	1/640	1/10	1/80	1/320	1/640
	118	1/10	1/40	1/640	1/1280	-	1/40	1/1280	1/2560	1/10	1/160	1/10240	1/10240
	34	1/20	1/20	1/80	1/80	1/20	1/40	1/5120	1/640	1/20	1/40	1/5120	1/1280
	56	-	1/10	1/320	1/160	1/10	1/40	1/2560	1/640	1/20	1/40	1/2560	1/2560
	24	1/20	1/40	1/320	1/320	1/30	1/60	1/5120	1/640	1/10	1/20	1/5120	1/5120
	94	1/40	1/40	1/1280	1/5120	1/40	1/40	1/5120	1/2560	1/20	1/40	1/10240	>1/20480
	97	1/20	1/40	1/80	1/160	1/10	1/40	1/1280	1/640	1/40	1/40	1/2560	1/2560
	3	1/20	1/20	1/80	1/160	-	1/40	1/160	1/160	1/80	1/80	1/2560	1/2560
	102	1/30	1/40	1/640	1/1280	1/160	1/80	1/2560	1/2560	1/10	1/30	1/1280	1/2560
FAVOURABLE	87	-	-	1/640	1/640	-	1/10	1/2560	1/2560	1/10	1/20	1/640	1/1280
	44	1/20	1/20	1/320	1/640	-	1/20	1/10240	1/2560	1/10	1/10	>1/20480	>1/20480
	68	-	1/20	1/320	1/320	1/20	1/20	1/5120	1/2560	1/10	1/20	1/2560	1/1280
	115	-	1/20	1/320	1/640	1/30	1/40	1/640	1/1280	1/15	1/20	1/1280	1/1280
	80	1/20	1/40	1/160	1/320	1/10	1/40	1/2560	1/640	-	1/40	1/2560	1/1280
	84	-	1/10	1/640	1/1280	1/20	1/40	1/2560	1/1280	1/10	1/80	1/7680	1/10240
	21	1/10	1/40	1/80	1/160	-	1/80	1/10240	1/2560	1/10	1/40	1/1280	1/1128
	103	1/10	1/80	1/320	1/640	1/15	1/80	1/2560	1/1280	-	1/160	1/1280	1/5120
	8	-	1/40	1/320	1/640	1/40	1/160	1/10240	1/10240	1/20	1/80	1/10240	>1/20480
	111	1/40	-	1/160	1/640	1/80	1/160	1/640	1/1280	1/10	1/40	1/2560	1/5120
	72	-	1/640	1/2560	1/1280	1/10	1/2560	1/5120	1/5120	1/20	1/1280	>1/20480	>1/20480
	53	1/20	1/160	1/1280	1/1280	1/60	1/1280	1/5120	1/5120	1/20	1/320	1/7680	1/5120
	67	1/80	1/320	1/2560	1/1280	1/160	1/960	>1/20480	>1/20480	1/80	1/240	>1/20480	>1/20480

Different degraded blue colours from white to marine blue define the six categories: negative (-), very low response (<1:20), low to moderate response (1:20 to<1:40), moderate response (1:40 to<1:80), high positive response (1:80 to<1:160), and strong positive response (≥1:160).

Overall, higher NAb titers were consistently observed against the Margarita strain (genotype 1.4). By day 21 post-vaccination, although NAb responses were predominantly higher against the Margarita strain, comparably high titers were also detected against the Diepholz strain (genotype 2.3), indicating a broad neutralizing activity across genetically distinct CSFV strains.

**Figure 3:**
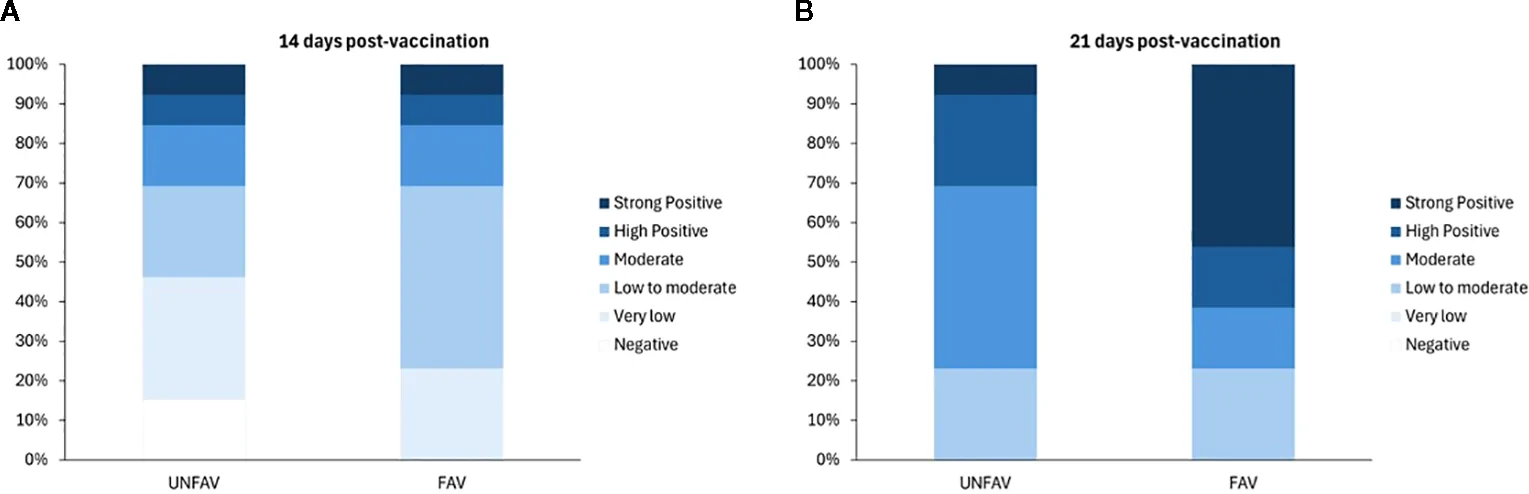
Histograms showing the distribution of neutralizing antibody response (%) in the genetically divergent favourable (FAV) and unfavourable (UNFAV) pig groups at 14 days post-vaccination **(A)** and 21 days post-vaccination **(B)**.

Based on the predefined NAb intensity categories, animals were initially compared using a low to moderate response threshold (≥1:20). At 14 days post-vaccination, significant differences between groups were observed: 46% of animals in the UNFAV group showed negative or very low neutralizing antibody response against any CSFV strain, compared with 23% in the FAV group (**Figure 3A**).

By 21 days post-vaccination, all animals exhibited at least a low to moderate neutralizing antibody response (≥1:20) to CSFV (**Table 3**). However, when applying a higher threshold corresponding to a high positive response (≥1:80), significant differences between groups persisted. At this time point, 61% of animals in the FAV group had developed high or strong positive neutralizing antibody titers, mainly against the Margarita and Diepholz strains, whereas only 31% of animals in the UNFAV group reached this response level (**Figure 3B**).

At 28 days post-vaccination, one week after administration of the second vaccine dose, all animals in both groups exhibited a strong neutralizing antibody response (≥1:160) against CSFV, at least against the Diepholz (CSFV genotype 2.3) and Margarita (CSFV genotype 1.4) strains (**Table 3**).

**Figure 4:**
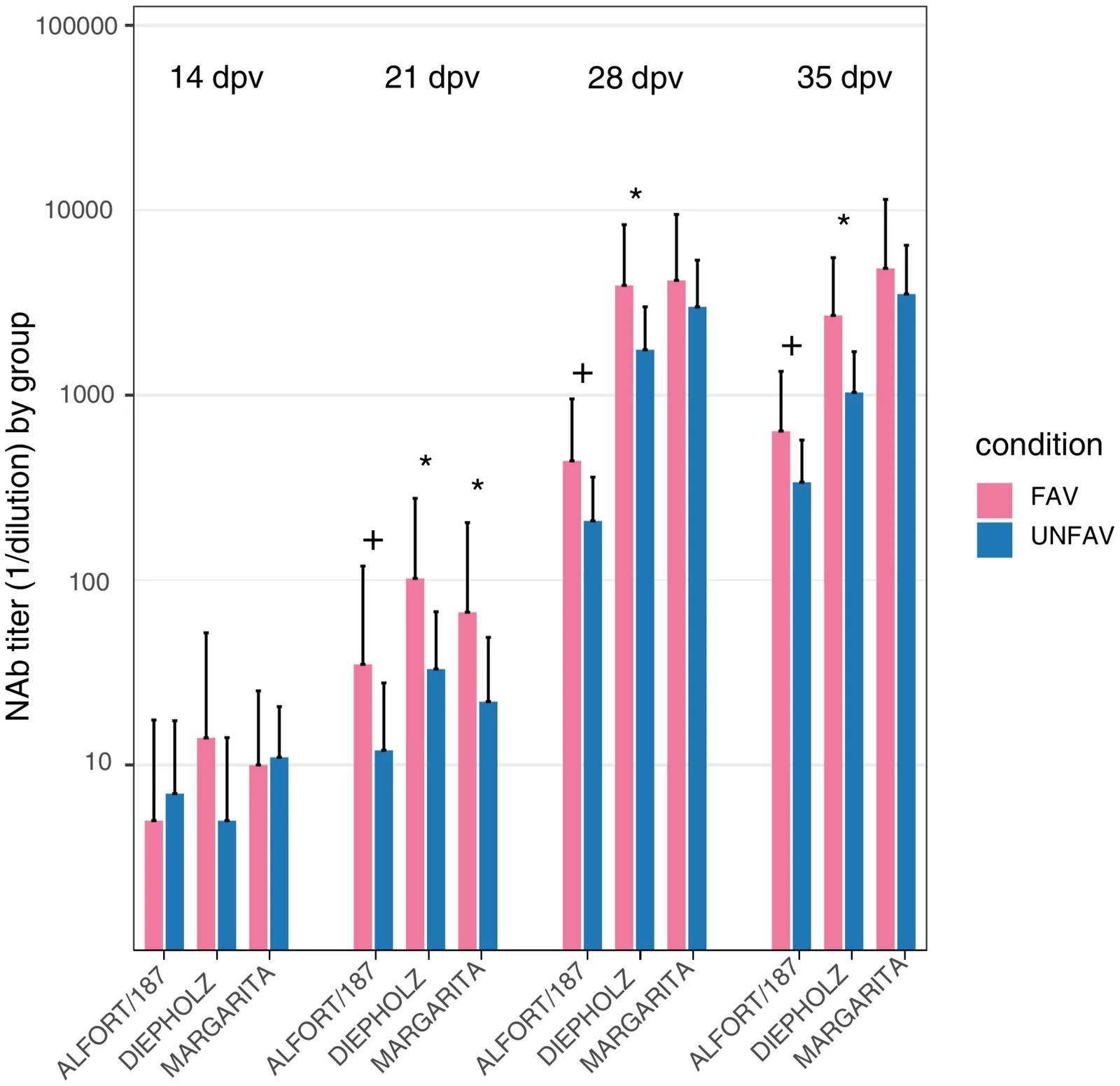
Neutralizing antibodies titers according to genetic group condition. Bars represent the geometric mean of the antibody titers plus the 95% confidence interval. *P < 0.05; ^+^*P* < 0.1.

The geometric mean of the titers (GMT) against the three CSFV strains showed significant differences between the FAV and UNFAV groups at 21 days post-vaccination, with the FAV group exhibiting higher NAb titers compared to the UNFAV group (**Figure 4**). No significant differences in the NAb titers against CSFV Margarita strain were observed among groups after two vaccine administrations. However, differences among groups in NAb titers against Alfort/187 and Diepholz strains persisted at both 28- and 35-days post-vaccination (**Figure 4**).

### Genetic markers associated to neutralizing antibody response

**Figure 5:**
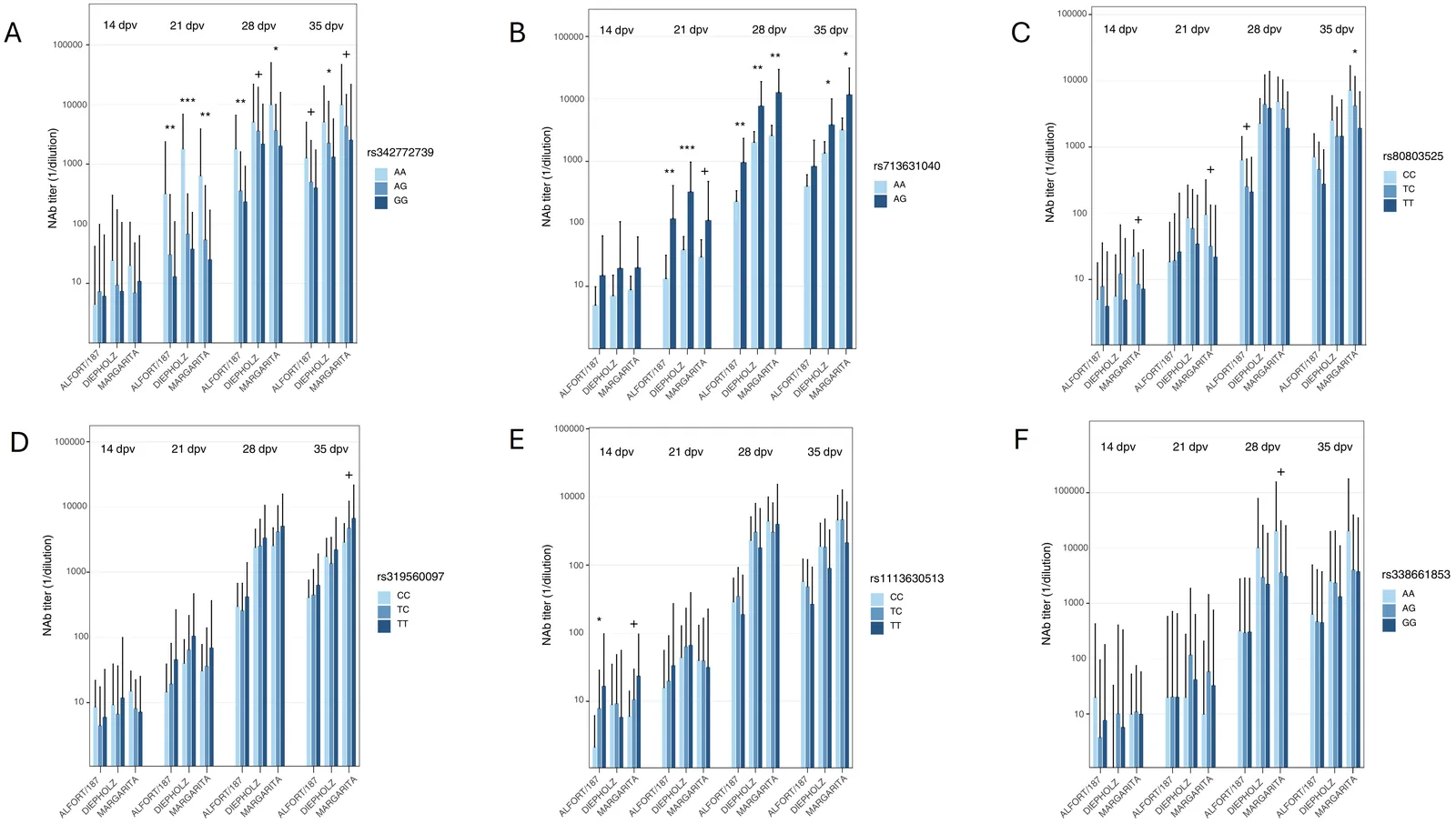
Neutralizing antibodies titers according to genotype for SNPs **(A)** rs342772739, **(B)** rs713631040, **(C)** rs80803525, **(D)** rs319560097, **(E)** rs1113630513, and **(F)** rs338661853. Bars represent the geometric mean of the antibody titers plus the 95% confidence interval. Statistical significance is indicated as follows: ***P < 0.001; **P < 0.01; *P < 0.05; ^+^*P* < 0.1.

Among all evaluated genetic markers, the SNP rs342772739 showed the strongest association with neutralizing antibody response. At 21 days post-vaccination, all animals carrying the AA genotype displayed a strong neutralizing antibody response against all three CSFV strains, reaching NAb titers ≥1:160 (animals 53 and 72 in **Table 3**). Although NAb titers increased significantly following the second vaccination in all genotypes (**Figure 5A**), marked differences persisted among them. In particular, animals with the AA genotype reached the highest GMT values, clearly exceeding those observed in AG and GG animals at days 28 and 35 post-vaccination.

Another SNP strongly associated with seroneutralization was rs713631040. Although no individuals with the GG genotype were identified, 60% of animals carrying the AG genotype displayed a robust NAb response against all three CSFV strains, reaching titers ≥1:160 at 21 days post-vaccination (animals 24, 44, 53, 67 and 72 in **Table 3**). Following the second vaccination, NAb titers against the three CSFV strains increased significantly in both genotypes, with marked differences in GMTs between genotypes for each strain (**Figure 5B**).

The C allele of rs80803525 was also significantly related to seroneutralization. At 14 days post-vaccination, 33% of CC animals had already developed a very positive NAb response (≥1:80) (animals 3, 67, 72, 84, 87 and 112 in **Table 3**), whereas none of the TT animals showed detectable neutralization (≤1:40) (animals 21, 56, 68, 73 and 103 in **Table 3**). At 21 days, the GMT of CC animals (1:96) was significantly higher than those of TC (1:32) and TT (1:22) genotypes for the Margarita strain (*P* < 0.1) (**Figure 5C**). These genotype-dependent differences remained significant after the second dose administration for the Alfort/187 and Margarita strains at days 28 and 35 post-vaccination (**Figure 5C**).

The T allele of rs319560097 showed a moderate association with seroneutralization. However, significant differences in NAb response between genotypes were only observed against Margarita strain at 35 days post-vaccination, with TT animals reaching a GMT above 1:6754, whereas CC animals showed a GMT of 1:2898 (*P* < 0.1) (**Figure 5D**).

The T allele of rs1113630513 was significantly correlated with higher seroneutralization at 14 days post-vaccination against Margarita and Alfort/187 strains (**Figure 5E**). Following administration of the second vaccine dose, this association was reversed, and animals carrying the C allele tended to have stronger NAb responses, although no statistically significant differences were observed (**Figure 5E**).

Finally, the SNP rs338661853 was not associated with seroneutralization neither at 14-days nor at 21-days post-vaccination (**Figure 5F**). Following the second vaccination, results suggest certain superiority of A allele, with the unique animal AA (animal 44 in **Table 3**) reaching a GMT > 1:20480 against the Margarita strain at 28 days post-vaccination *vs* a mean GMT of 1:2898 for the GG animals (**Figure 5F**).

## Discussion

Vaccination represents the most effective preventive tool available for numerous viral infections affecting humans and animals, with a substantial impact on infection rates, disease severity, hospitalizations, mortality, and economic losses (André, 2003). However, inter-individual variability in vaccine-induced immune responses is a key factor influencing the overall effectiveness of infectious disease control programs in pig production systems (Blanc et al., 2021). In the present study, we explore the association between several immunity-associated genetic markers and the humoral immune response elicited by the Porvac^®^ subunit vaccine against CSFV.

Porvac^®^ was used as an experimental model of subunit vaccination because it is highly effective at inducing robust CSFV-specific and neutralizing antibody responses and has demonstrated protective efficacy under field conditions against the lethal form of CSFV (Sordo-Puga et al., 2021, 2025). Specifically, we demonstrate that previously described genetic and immunological biomarkers of immunocompetence are associated with both the magnitude and the kinetics of vaccine-induced neutralizing antibody responses.

The selection of two genetically divergent groups enabled the identification of clear differences in early humoral immunity following vaccination. Pigs classified as genetically favourable exhibited significantly higher E2-specific antibody levels at 21 days post-vaccination and a higher proportion of animals with detectable neutralizing activity as early as 14 days post-vaccination, following a single vaccine dose. These early differences are particularly relevant from a disease control perspective, as the rapid development of functional immunity is critical for limiting CSFV spread during outbreak situations or emergency vaccination campaigns (Ganges et al., 2020).

Although both groups mounted strong antibody responses following the second vaccine dose, the superior early response observed in the favourable group supports the hypothesis that host genetic background influences the efficiency of primary immune activation. Importantly, the observed differences relate to the timing and magnitude of the response rather than to the overall capacity of the vaccine to induce protective immunity. The use of Porvac^®^ as a subunit vaccine model therefore allowed the assessment of genetic effects on immune responsiveness in a context where vaccine-induced humoral immunity is known to be strong and protective, minimizing confounding effects related to insufficient immunogenicity. Our findings highlight the potential of host-directed strategies to optimize vaccine performance by accelerating the onset of protective immunity through genetic selection, while preserving the advantages of subunit vaccines in terms of biosafety and DIVA compatibility (reviewed in Kamboj et al., 2024).

In this context, neutralizing antibodies directed against the E2 glycoprotein are considered a major correlate of protection against CSFV. Previous studies have demonstrated that Porvac^®^ confers robust clinical and virological protection against virulent CSFV challenge, including the prevention of transplacental transmission, and induces neutralizing antibodies capable of neutralizing genetically distinct CSFV strains belonging to different genotypes. This broad neutralizing activity supports the induction of broadly cross-reactive humoral immunity and reinforces the biological relevance of the differences observed in the magnitude and kinetics of vaccine-induced neutralizing antibody responses. Therefore, host genetic factors associated with enhanced early neutralizing responses may contribute to a more rapid establishment of protective immunity following vaccination, potentially improving vaccine performance under outbreak conditions (Muñoz-González et al., 2017; Sordo-Puga et al., 2021; Puente-Marin et al., 2025).

Among the genetic markers evaluated, the SNP rs342772739 showed the strongest association with neutralizing antibody responses. This marker had previously been associated with the abundance of peripheral blood γδ T cells and also with blood mRNA levels of *SOX13* (Ballester et al., 2020; Tarrés et al., 2024), a gene that encodes a transcription factor essential for γδ T cell development (Melichar et al., 2007). The consistently higher neutralization titers observed in animals carrying the AA genotype, associated with the higher proportion of γδ T-cell abundance and *SOX13* expression, suggest a relevant role for γδ T cells in shaping early vaccine-induced humoral immunity against CSFV. Furthermore, although all genotypes responded to the second vaccine dose, the magnitude of the neutralizing antibody response appeared to be strongly influenced by baseline γδ T-cell levels determined by this SNP.

These data support a functional link between γδ T-cell abundance and vaccine-induced humoral immunity, with the AA genotype of rs342772739 conferring a clear immunological advantage. In line with this interpretation, enhanced recruitment or availability of γδ T cells during early-life vaccination has been associated with improved vaccine efficacy in animals (reviewed in Le Page et al., 2022). Consistently, TRDC-knockout pigs lacking γδ T cells exhibited reduced neutralizing antibody titers following vaccination against CSFV using a highly attenuated live virus (Petersen et al., 2021).

In addition, the G allele of SNP rs713631040 was significantly associated with stronger neutralizing antibody responses. This allele had been previously associated with lower serum CRP concentrations (Hernández-Banqué et al., 2023). This association suggests that a lower basal inflammatory status may favour more efficient vaccine-induced immune activation, with the G allele promoting enhanced antibody responses at later time points post-vaccination.

Interestingly, the C allele of rs80803525, previously associated with reduced peripheral lymphocyte counts (Ballester et al., 2020), was also linked to enhanced neutralizing responses. In animals of the same age and genetic background, Ballester et al. (2020) reported negative genetic correlations between total lymphocyte counts and the relative abundance of B lymphocytes, T helper cells and γδ T cells, together with a positive correlation with cytotoxic T lymphocytes. Accordingly, although initially counterintuitive, this association suggests that animals with lower total lymphocyte counts may display a lymphocyte subset composition more favourable for mounting an efficient vaccine-induced humoral response.

A particularly noteworthy finding was the dynamic association observed for SNP rs1113630513, which had been previously related to the relative abundance of memory T-cells. Genotypes associated with lower memory T-cells abundance showed stronger early neutralizing responses, whereas genotypes linked to higher memory T-cell levels exhibited superior responses following the second vaccine dose. This temporal shift suggests differential genetic control of primary and secondary immune responses and underscores the importance of considering response kinetics when evaluating genetic associations with vaccine-induced immunity.

Finally, the markers previously associated in Ballester et al. (2020) with plasma IgG levels (rs319560097) and lymphocyte phagocytic capacity (rs338661853) showed partial or non-significant associations with virus neutralization. These findings suggest that baseline IgG concentrations may reflect general immunocompetence but are not sufficient predictors of antigen-specific or functionally protective antibody responses. Furthermore, the limited number of animals carrying the A allele of rs338661853 constrained definitive conclusions regarding this SNP.

Overall, our results suggest that immune responses to CSFV vaccination are under polygenic control and involve genetic determinants linked to both innate and adaptive immunity. The evaluation of this biomarker panel in a CSFV subunit vaccine model reinforces its potential applicability in breeding programs aimed at improving immunocompetence and vaccine responsiveness in pigs. Nevertheless, several aspects warrant further investigation. Although the number of vaccinated animals was sufficient to detect consistent genetic associations, larger and more diverse populations will be required to assess the robustness and transferability of these findings across breeds and production systems. In addition, while the present study focused on humoral immune responses, the integration of protection outcomes following experimental challenge, together with in-depth analyses of antigen-specific cellular immunity, will be critical to fully elucidate the functional relevance of the identified biomarkers. Such approaches will not only strengthen the biological interpretation of host genetic effects on vaccine responsiveness but also support the translation of these biomarkers into predictive tools for breeding strategies aimed at enhancing immunocompetence and disease resilience in pigs. Finally, future studies should also evaluate the impact of these genetic markers on productive traits, particularly to identify potential trade-offs between health and production traits.

## Conclusion

Our results demonstrate that host genetic variation significantly shapes vaccine-induced immunity against CSFV. The identified genetic markers represent promising tools for integrating immunocompetence into pig breeding programs, with the potential to enhance vaccine responsiveness, improve population robustness, and support sustainable disease control in pig production systems.

## Data Availability

The original contributions presented in the study are included in the article/**Supplementary Material**, further inquiries can be directed to the corresponding author/s.
