# Supplementary material for: Porcine genetic markers of immunocompetence shape the immune response to a classical swine fever virus subunit vaccine
Source: Front Cell Infect Microbiol. 2026 Aug 11;16:1803004. doi: 10.3389/fcimb.2026.1803004 (PMC13503205; doi:10.3389/fcimb.2026.1803004)
Supplement: Supplementary file 1 [file Table1.docx]

Supplementary Material

**Supplementary Table 1**. Ingredient and nutrient composition of the pig diet.

| Ingredients | % |
| --- | --- |
| Maize | 32.0 |
| Wheat | 19.32 |
| Barley | 18.0 |
| Soybean meal | 16.29 |
| Sunflower meal | 3.69 |
| Fat | 2.68 |
| Biscuit meal | 2.0 |
| Beet Pulp | 2.0 |
| Celatom | 1.0 |
| L-Lysine (liquid) | 0.73 |
| Calcium Carbonate | 0.69 |
| Monocalcium Phosphate | 0.43 |
| QBT Pigs PHY xb | 0.4 |
| Salt | 0.39 |
| L-Threonine | 0.16 |
| Toxo-Mx | 0.1 |
| DL-Methionine | 0.10 |
| L-Tryptophan | 0.02 |
|  |  |
| Calculated Nutrient Composition |  |
| Net Energy (kcal/kg) | 2,475.00 |
| Crude Protein (%) | 16.0 |
| Crude Fibre (%) | 4.10 |
| Fat (%) | 4.85 |
| SID Methionine (%) | 0.32 |
| SID Methionie+Cistine (%) | 0.59 |
| SID Lysine (%) | 1.00 |
| SID Threonine (%) | 0.63 |
| SID Tryptophan (%) | 0.18 |
| SID Arginine (%) | 0.88 |
| SID Valine (%) | 0.64 |
| Calcium (%) | 0.70 |
| Total Phosphorus (%) | 0.46 |
| Digestible Phosphorus (%) | 0.32 |
